# The Accuracy and Precision of Memory for Natural Scenes: A Walk in the Park

**DOI:** 10.1162/opmi_a_00122

**Published:** 2024-03-01

**Authors:** Leo Westebbe, Yibiao Liang, Erik Blaser

**Affiliations:** Department of Psychology, University of Massachusetts Boston, Boston, MA, USA

**Keywords:** scene memory, visual perception, continuous report, delayed estimation, similarity advantage, boundary extension, natural scenes, route loop

## Abstract

It is challenging to quantify the accuracy and precision of scene memory because it is unclear what ‘space’ scenes occupy (how can we quantify error when misremembering a natural scene?). To address this, we exploited the ecologically valid, metric space in which scenes occur and are represented: routes. In a delayed estimation task, participants briefly saw a target scene drawn from a video of an outdoor ‘route loop’, then used a continuous report wheel of the route to pinpoint the scene. Accuracy was high and unbiased, indicating there was no net boundary extension/contraction. Interestingly, precision was higher for routes that were *more* self-similar (as characterized by the half-life, in meters, of a route’s Multiscale Structural Similarity index), consistent with previous work finding a ‘similarity advantage’ where memory precision is regulated according to task demands. Overall, scenes were remembered to within a few meters of their actual location.

## INTRODUCTION

Is scene memory good or bad? Over the past 100 years, work in scene memory has had a familiar character: participants are shown a series of to-be-remembered images, then asked to pick them out from among novel foils (Strong, 1912). Capacity estimates for even briefly shown scenes (Potter & Levy, 1969), even over long retention intervals (Shepard, 1967), are remarkably high, famously reaching 10,000 images (Standing, 1973). While certainly *immense*, it is not clear if this figure is *impressive*, as there is no obvious way to characterize the difficulty of the task or quantify the accuracy and precision of the memories themselves.

Part of the challenge is that scene memory could be based on multiple sources of information (Bainbridge et al., 2019; Malcolm et al., 2016), including image-based gist (Brady et al., 2017; Cunningham et al., 2015; Greene & Oliva, 2009), higher-level schematic / ‘semantic’ knowledge about the presence and arrangement of objects and surfaces (Biederman, 1972; Hock & Schmelzkopf, 1980; Konkle et al., 2010b; Velisavljević & Elder, 2008; Võ, 2021), and details about constituent objects themselves (Brady et al., 2008; Hollingworth, 2004; Konkle et al., 2010a). Problematically, it is not clear how to define the ‘space’ scenes occupy, so there is no obvious metric to quantify accuracy and precision. One way to address this is to *create* a space through a parameterized manipulation of scene characteristics. Recent work has used machine learning, specifically deep generative models, to generate sequences of complex, naturalistic scenes, spanning a chosen dimension (e.g., from *this* kitchen to *that* kitchen) (Kyle-Davidson et al., 2022). In a clever methodological advance, Son et al. (2022) showed how these stimuli could be employed similarly to basic visual feature manipulations in formal tests of visual memory. Utilizing their ‘scene wheels’ in a delayed estimation, continuous report paradigm allowed them to quantify the accuracy and precision of scene memory, in the units of the generative model’s scene space. The present study builds on these approaches, but instead of creating a space, takes advantage of an ecologically valid, metric one within which all scenes occur: routes.

### Scenes-in-routes

It is well-known that the statistics of natural scenes (Ruderman & Bialek, 1994; Tkačik et al., 2011) are reflected in, and exploited by, the organization of the visual system (Baddeley et al., 1997; Barlow & Rosenblith, 1959; Field, 1987; Geisler, 2008; Simoncelli & Olshausen, 2001). This is true also of the higher-level ‘semantic’ structure (Võ, 2021) of everyday scenes composed of recognizable objects (for a review, see Kaiser et al., 2019). Beyond encoding and recognition, memory respects these constraints as well, with, for instance, scenes with typical schematics being better remembered than random assemblages (Castelhano & Krzyś, 2020; Mandler & Parker, 1976; Mooney, 1960).

Scenes exist within routes; an unavoidable consequence of a visual system moving through the environment (Gibson, 1950; Koenderink, 1986). Scenes drawn from routes can be readily identified, distinguished from scenes that do not belong, and placed in highly accurate distance relationships to one another (Allen et al., 1978; Jenkins et al., 1978). Indeed, when viewing a sequence of scenes drawn from a route, the observer is already predicting the characteristics of upcoming scenes (Cornell et al., 1999; Smith & Loschky, 2019), exploiting serial dependencies (for a review, see Pascucci et al., 2023). Participants shown a sequence of scenes (Hock & Schmelzkopf, 1980; Moar & Carleton, 1982) or led or driven along a route (Gärling et al., 1981; Ishikawa & Montello, 2006) and then later shown two target scenes can make accurate, consistent judgments about their direction and distance from one another. The fact that this is true even if the scenes that comprise the route are presented in a shuffled, random order (Allen et al., 1978) has been taken as evidence that participants both leverage information from landmarks to help organize scenes, and also place them within schemas acquired during development (Herman & Siegel, 1977)^1^.

The fundamental importance of scenes-in-routes is reflected in the visual brain (Kamps et al., 2016), where the parahippocampal place area (Epstein & Kanwisher, 1998) and retrosplenial complex have been implicated in both memory for scenes and identification of landmark objects in the context of a route (Epstein & Vass, 2014). These systems also retain plasticity, adapting to increased demand, famously evidenced by the gray matter volume increase of the posterior hippocampi of successfully trained London taxi drivers (Woollett & Maguire, 2011). And, these spatial relationships are not just relative, but *metric* with, for instance, hippocampal activity reflecting distances between scenes along a route (Morgan et al., 2011). This all makes sense of course: for an active, embodied visual system, contextualizing and exploiting scenes-in-routes to inform a cognitive map of the environment facilitates visual navigation (Epstein et al., 2017; Rolls, 2023; Thorndyke & Hayes-Roth, 1982; Zeil, 2023)^2^. Taken together then, the spatiotemporal dependencies of scenes as a function of distance traveled (Calow & Lappe, 2007; Hyvärinen et al., 2009; van Hateren & Ruderman, 1998) offer a metric, ecologically valid space in which to situate scene memory.

## METHODS

### Overview

**Figure 1.**
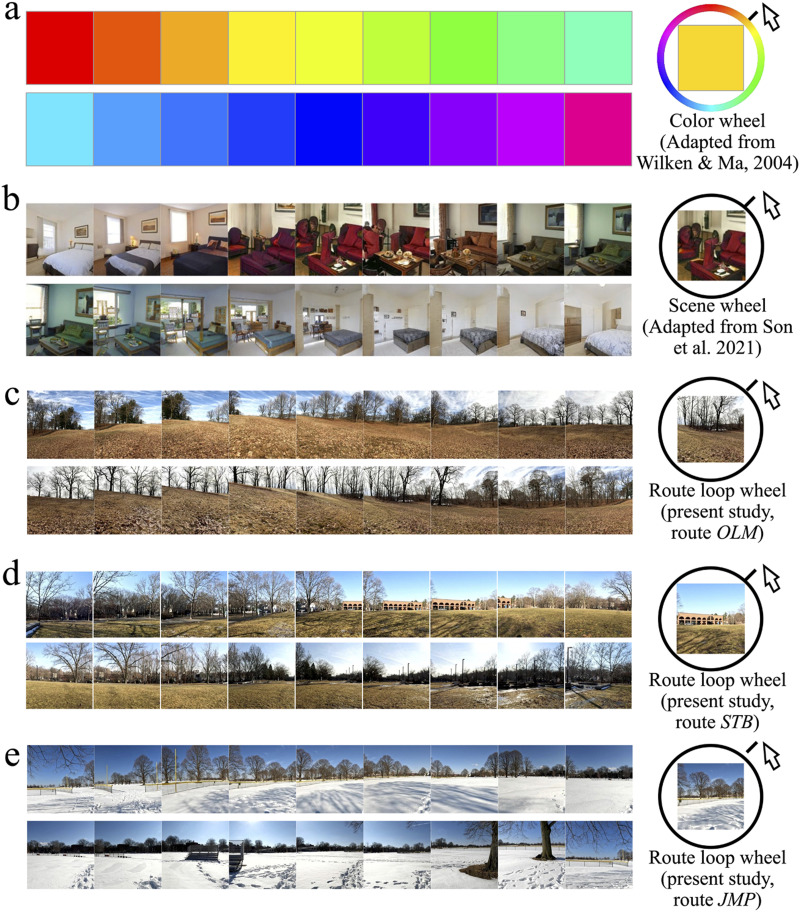
Examples of stimulus spaces. A continuous response wheel in the context of a delayed estimation task allows the participant to pinpoint their memory within a given space, for instance a CIE color space (a), a machine learning generated space of bedrooms (b), or, in our case, an outdoor ‘route loop’ (c–e). The relevant ‘units’ are determined by the space: CIE coordinates, increments within the latent space of the GAN, and meters of travel, respectively. In the route loop examples shown here (routes OLM, STB, and JMP, respectively), scenes are separated by 5 m (20 deg of travel around the route loop). In the actual experiment, the response wheel moved between scenes separated by 0.25 m. We found that most scenes were remembered to within ∼3 m of their actual location.

Researchers studying visual memory have used a continuous report, delayed estimation task that allows for a high level of parametric control, facilitating the measurement of accuracy and precision of memory for basic features such as orientation, spatial frequency, and color (Wilken & Ma, 2004). Typically in these studies, participants are shown a to-be-remembered target stimulus and then provided a continuous response ‘wheel’ on which to pinpoint their memory for what they had been shown (Figure 1A). For basic feature spaces, the dimensions circumscribed by the wheel have considerable face validity, like the 360 degrees of rotation an object can exhibit in a 2D plane or a CIE color space. Son et al. (2022) extended this approach by using machine learning to generate ‘scene wheels’ within a space of, say, bedrooms, such that neighboring steps on the wheel are maximally similar, and rooms separated by 180 degrees maximally dissimilar (Figure 1B). And, just as in a color space, they could vary the radius of the wheel to manipulate the range of stimuli presented and the increments between neighboring stimuli. In all of these types of studies, performance is quantified in terms of error, that is, the ‘distance’ between the target stimulus and the chosen response. For orientation and color, accuracy and precision can be characterized in the natural units of the space, like degrees of rotation or CIE coordinates. Within, say, a bedroom space, there is no natural unit, so performance can be quantified using increments of the response wheel itself (i.e., degrees of error) (Son et al., 2022). In the present study, our main goal was to characterize the accuracy and precision of scene memory within the pre-existing, ecologically-valid space of routes. Importantly, this space provides a natural unit that inherently governs the visual changes associated with moving through that space: meters (Figure 1C–E).

In the present delayed estimation study, from trial-to-trial, participants were briefly shown a target scene from an outdoor route and then used a continuous report wheel to scrub through the route to pinpoint their memory of the scene. The circumferences of the routes were kept constant, at 90 m. However, three different routes were used, each of which inherently had differing levels of inter-scene ‘self-similarity’—a measure of visual change per meter traveled based on a Multiscale Structural Similarity analysis (Wang et al., 2003).

As described above, our overarching goal was to characterize the accuracy and precision of memory for scenes-in-routes. Beyond that, we had two hypotheses. First, we hypothesized that accuracy would be high (i.e., unbiased) and unrelated to route self-similarity. While there is an extensive literature on boundary extension (which would manifest in our study as a ‘zoomed out’ net backward bias; see Discussion), some recent work has challenged the ubiquity of the effect (Bainbridge & Baker, 2020; Lin et al., 2022), so we adopted the more conservative hypothesis of no net bias. Alongside this, we also tested for learning effects by tracking accuracy and precision across blocks, and over trials within a block. Second, we hypothesized that precision would be high, but less so for scenes drawn from more self-similar routes. For this latter hypothesis, however, we found the opposite relationship.

### Participants

Our experiment was pre-registered on OSF (Blaser & Westebbe, 2022) and hosted on *Cognition.run*, an online platform for delivering experiments. Prolific.co was used for recruitment and compensation. Online psychophysics can provide high-quality data (Semmelmann & Weigelt, 2017). The present experiment was especially well-suited to online testing because the task was straightforward and spatiotemporal requirements were modest (Anwyl-Irvine et al., 2021). Before the main experiment, each participant read through and agreed to an informed consent document. They then answered screening questions provided by Prolific.co to ensure fluency in English, normal or corrected-to-normal vision, and access to a recent computer operating system.

A total of 119 participants completed the study, 45 identified as female, the age range was from 18–58 (*M* = 26.9 years, *SD* = 8.7), and there was representation from 15 countries^3^. *Exclusion criteria*: After data collection, we applied our pre-registered exclusion criteria. Participants who had more than 25% invalid trials in any of the three (48 trial) blocks that comprised a testing session were excluded from all analyses. An invalid trial was one in which either no selection was made before the end of the 10 s *response period* for a trial (‘timeouts’) or the response was deemed an outlier (see Data Analysis below). 12 participants were excluded in this way, yielding a final sample of 107 participants. This sample size met the requirements of an a priori power analysis (G*Power) tailored to our pre-registered main analyses (one-way repeated measures ANOVA) assuming a medium effect size (*partial eta squared η*^2^ = 0.1), 0.8 power, and alpha 0.05.

### Route Loops

**Figure 2.**
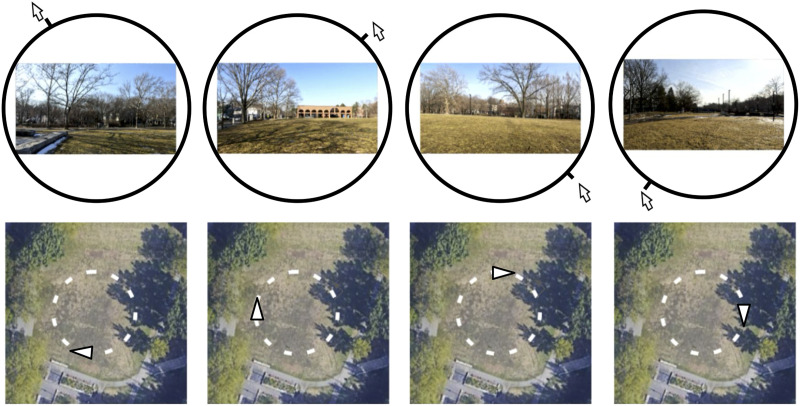
Moving the cursor around the continuous report wheel allows the participant to adjust their position along the route loop (route STB shown here). The overhead maps in the lower panels are shown here for reference; the white triangle indicates the position corresponding to the scene in the upper panels. From trial-to-trial the report wheel started at a random location.

First-person perspective videos were collected by author LW from various outdoor locations using a chest-mounted GoPro Hero 10 camera (using horizon leveling, image stabilization, and with a 75 × 42 deg FOV). At each location, LW staked out a 90 m circuit that started and ended in the same place. Using a metronome to maintain pace, LW walked (clockwise), for 60 s, at a typical walking speed of 1.5 meters per second (Franěk & Režný, 2017), yielding a *route loop*. A set of three route loops was culled from several alternate takes and locations. These three were named *OLM*, *STB*, and *JMP* after the parks in the Boston area where they were filmed (Olmstead Park, Stony Brook Park, and Joe Moakley Park, respectively). The locations were selected because they offered natural, open spaces, each with a distinct visual character. The routes, by design, contained primarily outdoor scenery and did not contain people, text, or dynamic elements. Selected routes were filmed under clear, unchanging weather conditions, at approximately 2 pm EST in late February–early March 2022. The three route loops were then exported at 6 fps, at 480 × 270, to create a set of 360 individual scenes (shared on OSF (Blaser & Westebbe, 2022), each spaced at approximately 0.25 m of travel, that would be used as stimuli and employed as response wheels in a continuous report paradigm (Figure 2).

### Procedure

Scripts controlling stimuli and response collection were written in jsPsych (de Leeuw, 2015), a JavaScript framework for behavioral experiments. Much of our code was tailored from scripts generously shared by Son et al. (2022). A testing session began with a set of instructions displayed on-screen, followed by 10 practice trials. Practice trials were identical to test trials, but used a unique route loop (collected on the University of Massachusetts Boston campus) and were not included in analyses. The main experiment consisted of three blocks of 48 trials, with each block corresponding to one of the three route loops, OLM, STB, or JMP. The order of the blocks was approximately counterbalanced across participants. Between blocks, participants were given a break (minimum 3 minutes), then began the next block with a keypress.

**Figure 3.**
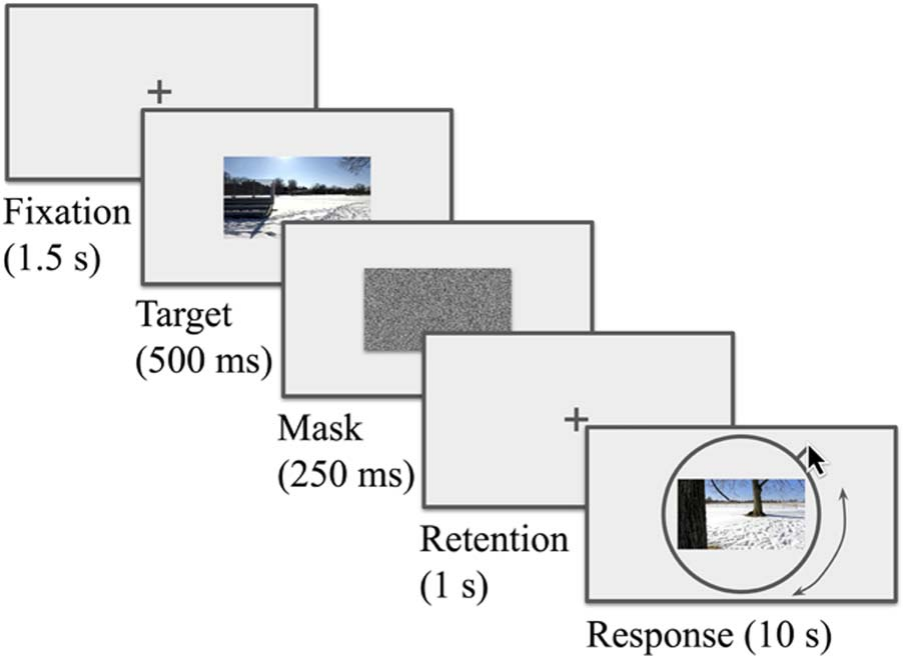
Typical trial. In each trial, participants were briefly shown a to-be-remembered target scene, then presented with a continuous report ‘wheel’. This wheel allowed participants to scrub through the corresponding route loop (JMP shown here) in an attempt to pinpoint the target scene. Each 90 m route loop was composed of 360 scenes, with each potential response spaced, then, at 0.25 m. In a series of three blocks, participants observed 48 scenes from the three routes (OLM, STB, and JMP).

Each trial started with a 1500 ms fixation cross. This was followed by a to-be-remembered *target scene*, i.e., a scene randomly drawn from the relevant route loop, displayed for 500 ms. The target scene was immediately followed by a 250 ms noise mask and a blank 1000 ms retention interval. The trial then entered the *response phase*. The participant could then use the mouse to travel forward or backward around the route loop (the starting scene was also chosen randomly among the 360 possibilities from trial-to-trial) (Figure 3). Participants were tasked with locating the *target scene* from memory. Ultimately, to indicate their selection, participants clicked the mouse button and the next trial began. If no selection was made within 10 seconds, the trial timed out, was marked as invalid, and the next trial began (time-outs were rare, see Data Analysis below). While we could not control viewing distance, on an average laptop screen (∼35 cm) at a typical arm’s-length viewing distance (∼57 cm), the target scene, mask, and response scene each subtended approximately 34 × 19 degrees of visual angle.

### Data Analysis

For each participant, we measured the accuracy and precision of memory for the target scenes. We also investigated the relationship between accuracy and precision and the self-similarity of the route (see Route Loop Self-Similarity below). We use the terms accuracy and precision here in their technical sense, as a measure of bias (net distance of the response from a target value) and dispersion of responses. For each trial, we measured *error* as the number of meters, ‘forward’ or ‘backward’, separating the chosen scene from the to-be-remembered target scene. The length of each route loop was 90 m and was composed of 360 individual scenes, so an error of 1 frame in our response wheel was equal to 0.25 m. Errors were always taken as the shortest path along the route loop from the response to the target scene (so, for instance, an error would be coded as −0.75 m (−3 frames) as opposed to +89.25 m (+357 frames)). If a trial ended in a time-out, the error value was left empty. In general, response times were well within the allotted 10 s for all three routes: OLM (*M* = 4.01 s, *SD* = 1.85), STB (*M* = 3.77 s, *SD* = 1.71 s), and JMP (*M* = 4.14 s, *SD* = 1.82), and time-outs were rare, occurring at a rate of 2.1%, 2.4%, and 2.1% for routes OLM, STB, and JMP respectively.

Prior to estimating accuracy and precision, we screened the set of responses from each participant for a particular block for outlier errors, defined as more than 3 median absolute deviations (MAD) away from the median (Leys et al., 2013). Outlier errors stem from two likely sources: lapses in attention, or *gross mislocalizations* of the target scene (e.g. a scene drawn from a stretch of a route containing say, a tree and a large grassy foreground, may be mislocalized as belonging to a disparate part of the route that may, by chance, contain similar visual elements). Consistent with our pre-registered plan, outlier errors were excluded from our main analyses and were set aside for secondary analyses.

After exclusions, to estimate a participant’s overall *accuracy* for a route, we took the median of the error values for the relevant block of trials. A negative median, then, would indicate a net backward bias toward scenes that preceded the target scene (a ‘zooming-out’ consistent with boundary extension), a positive median would indicate a net forward bias (a ‘zooming-in’ consistent with boundary contraction), and a near-zero median would indicate maximal accuracy and no bias. We hypothesized that accuracy would be unbiased (i.e., not significantly different from zero; centered on the true position of the scene within the route), and unrelated to the route self-similarity. To estimate a participant’s *precision* for a route, we took the median absolute deviation (MAD) of the error values in the relevant block. (MAD is a measure of dispersion, a robust analog to SD^4^, so decreasing MAD values indicate increasing precision, and vice versa.) We preferred these robust measures of central tendency and dispersion to sidestep strong assumptions about the parametric distribution of the error data and to mitigate the influence of large error values (Leys et al., 2013). We hypothesized that scene memory for routes with less self-similarity (more variability) would be remembered with greater precision. We found, however, that the opposite was true.

### Route Loop Self-Similarity

**Figure 4.**
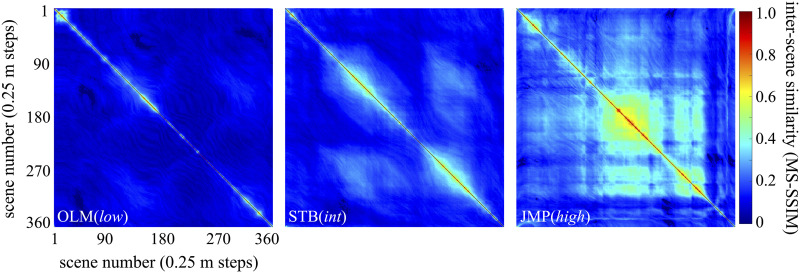
Heatmaps showing inter-scene self-similarity, based on the MS-SSIM index, for each of the three route loops. Each cell is a comparison between one of the 360 scenes in the route loop to one of the other scenes, and ranges from 0 (maximally dissimilar) to 1 (maximally similar; as will be the case for values along the diagonal). Heatmaps for the three scenes are presented in order, from left to right, of increasing self-similarity (OLM(*low*), STB(*int*), and JMP(*high*), respectively). While *half-life* (see below) was our main measure of route self-similarity, for reference the overall average of the MS-SSIM indices shown in the heatmaps is 0.09, 0.16, and 0.26, respectively.

**Figure 5.**
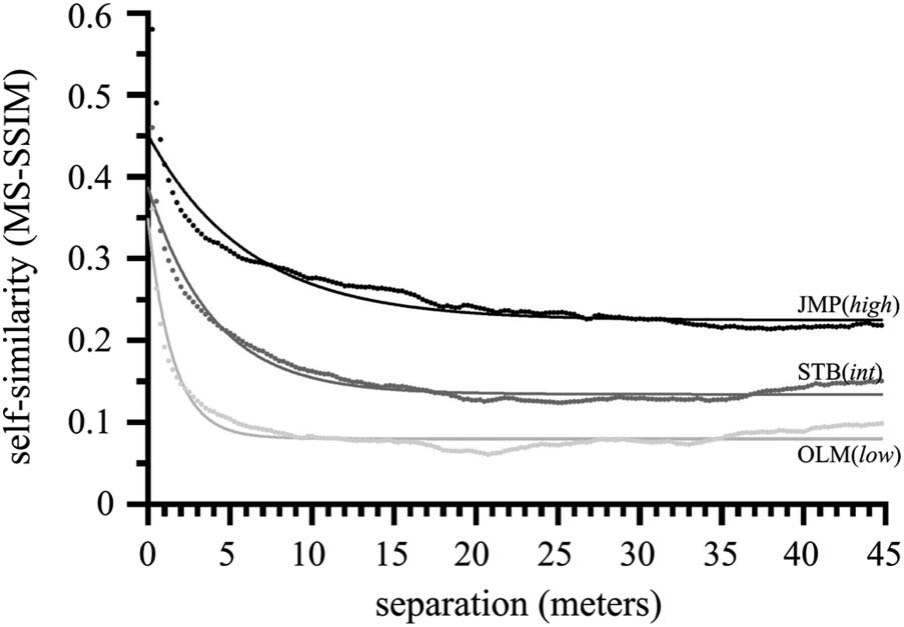
Inter-scene similarity (as measured by MS-SSIM) as a function of distance, i.e., on average for a particular route, how similar is a scene to one *X* meters (±45) away? These data are well fit by exponential decay (adjusted R^2^ of 0.89, 0.95, and 0.91 for routes OLM, STB, and JMP, respectively). As can be seen by the laminar decay curves, each route has a distinct overall inter-scene self-similarity. Based on decay rate, we used a route’s *half-life*, in meters, to characterize its self-similarity, with OLM having the lowest (half-life of 1.05 m), STB intermediate (2.75 m), and JMP highest (4.24 m).

To characterize the ‘self-similarity’ of a route, we used the Multiscale Structural Similarity (MS-SSIM) index. MS-SSIM models aspects of the human visual system, analyzing luminance, contrast, and structural information at various scales in order to approximate perceptual judgments of similarity (Wang et al., 2003). MS-SSIM has been widely validated and outperforms simpler measures based on pixel-wise differences between images (Rouse & Hemami, 2008; Snell et al., 2017). We determined the MS-SSIM index between each scene and every other scene within a route, as a function of their separation (Figure 4). In this implementation (MATLAB 2022a), the index ranges from 0 (maximally dissimilar images) to 1 (identical images). The mean of these resulting similarity values for separations from 0.25 m (the minimum distance possible, corresponding to neighboring scenes) to 45 m (the maximum possible distance along the route, corresponding to ±180 deg along the route wheel) is shown in Figure 5. As can be seen, for all three routes, inter-scene similarity falls off quickly as a function of separation and is well-captured by exponential decay (adjusted R^2^ of 0.89, 0.95, and 0.91 for routes OLM, STB, and JMP, respectively, with AIC probabilities giving >99.99% probability for exponential decay model versus <0.01% relative probability for a null of linear fit). Critically for our purposes, a nonlinear (exponential decay) regression confirmed that the three routes had significantly *different* self-similarities (the AIC probability that the three curves differed was >99.99% versus a relative probability of <0.01% for the null that there was just one curve for all three data sets, and a further test showed that the AIC probability that the *decay rate* itself differed was >99.99% versus a relative probability of <0.01% for the null that the three curves shared the same decay).

We could then quantify a route’s self-similarity by its *half-life* (*ln*(2)/*decayRate*). Half-life is a useful characterization here because it is in a natural unit, *meters*, and reflects the rate of change in similarity as a function of distance; as one walks along the route, how rapidly does the scenery change? A route, then, comprised of scenes that have, on average, long half-lives is relatively self-similar, while one comprised of scenes with relatively short half-lives tends to exhibit rapid visual change with distance traveled. Similarity dropped by half after traveling a distance of 1.05 m, 2.75 m, and 4.24 m, along routes OLM, STM, and JMP, respectively. Based on this, we could rank the three routes, with route OLM having relatively *low* self-similarity, STB *intermediate*, and JMP relatively *high* self-similarity^5^. For clarity moving forward, we will refer to these routes as OLM(*low*), STB(*int*), and JMP(*high*).

## RESULTS

### Block Order Effects

In a testing session, participants ran three blocks, one per route, of 48 trials. Here we tested for potential learning effects by assessing sequential effects across the three blocks. First, we conducted a one-way repeated measures ANOVA to assess the effect of block order on the *accuracy* of memory for a route. As described above, the measure for accuracy was the median of the response errors, in meters, for each participant and block. We found that accuracy was very high (i.e., near zero median error) and there was no significant effect of order *F*(2, 212) = 1.13, *p* = 0.32, *partial eta squared η*^2^ = 0.012, suggesting that the accuracy of memory for scenes was not affected by position, i.e., whether a block was tested as the 1st (*M* = 0.07 m, *SD* = 0.72 m), 2nd (*M* = 0.14 m, *SD* = 0.67 m), or 3rd (*M* = 0.004 m, *SD* = 0.71 m) within the session. We then conducted a one-way repeated measures ANOVA to measure the effect of block order on memory *precision*. As described above, the precision was measured as the median absolute deviation (MAD) of the response errors for each participant and block, expressed in meters. As a measure of dispersion, higher MAD values indicate lower precision and lower MAD values higher precision. Again, we did not find a significant effect of block order *F*(2, 212) = 1.96, *p* = 0.143, *η*^2^ = 0.018, i.e., no difference in precision whether a block was tested as the 1st (*M* = 2.93 m, *SD* = 3.31), 2nd (*M* = 3.12 m, *SD* = 3.82), or third (*M* = 2.50 m, *SD* = 1.64 m) block within the session.

### Trial Order Effects

Here we sought to test for learning effects by assessing sequential effects across trials within a block. To do this, we collapsed data across participants for each of the 48 trials for a route. We then performed a linear regression on both accuracy and precision as a function of trial. This regression analysis found no effect of trial on accuracy, with the regression slopes for each of the three routes not significantly different from zero, OLM(*low*): *F*(1, 46) = 0.788, *p* = 0.379, R^2^ = 0.017; STB(*int*): *F*(1, 46) = 1.32, *p* = 0.26, R^2^ = 0.028; JMP(*high*): *F*(1, 46) = 0.158, *p* = 0.69, R^2^ = 0.003. The regression analysis also found no effect of trial on precision, with the regression slopes for each of the three routes not significantly different from zero, OLM(*low*): *F*(1, 46) = 2.32, *p* = 0.13, R^2^ = 0.048; STB(*int*): *F*(1, 46) = 0.04, *p* = 0.85, R^2^ = 0.0008; and JMP(*high*): *F*(1, 46) = 0.64, *p* = 0.43, R^2^ = 0.014).

### Scene Memory Accuracy

**Figure 6.**
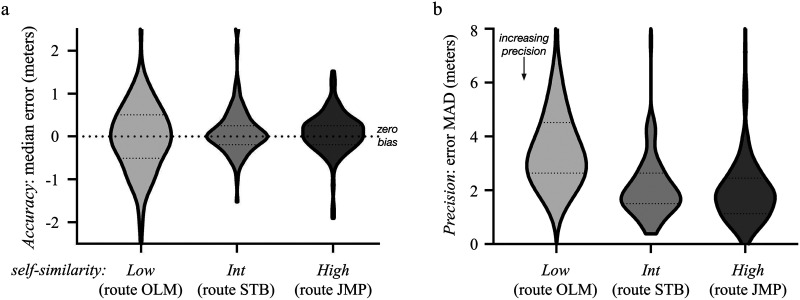
Violin plots (truncated, with iqr indicated with dotted lines) of scene memory accuracy and precision (*N* = 107). (a) Scene memory *accuracy*: average median response error for each participant, for each of the three routes, ranked in terms of route self-similarity: OLM(*low*) (*M* = −0.01 m, *SD* = 0.86), STB(*int*) (*M* = 0.16 m, *SD* = 0.64), and JMP(*high*) (*M* = 0.06 m, *SD* = 0.56). (b) Scene memory *precision*: average MAD (median absolute deviation) of the response errors for each participant, for each of the three routes, ranked in terms of route self-similarity: OLM(*low*) (*M* = 4.09 m, *SD* = 3.70), STB(*int*) (*M* = 2.40 m, *SD* = 3.18), and JMP(*high*) (*M* = 2.06 m, *SD* = 1.52).

To quantify scene memory accuracy, and the potential influence of route self-similarity, we performed a one-way repeated measures ANOVA, with accuracy (median of the response error distribution, in meters) for each participant as the dependent variable and with route self-similarity (*low*, *int*, and *high*) as the factor. As expected, there was no significant effect of route, *F*(2, 212) = 1.71, *p* = 0.18, *η*^2^ = 0.016. Also as expected, accuracy was high, i.e. average median error was indistinguishable from zero, for each of the routes, OLM(*low*): *M* = −0.01 m, *SD* = 0.86, *p* = 0.998; STB(*int*): *M* = 0.16 m, *SD* = 0.64, *p* = 0.129; JMP(*high*): *M* = 0.06 m, *SD* = 0.56, *p* = 0.757, corrected for multiple comparisons with Dunnett’s method. These results show route self-similarity had no significant effect on accuracy and that there was no net bias, forward or backward (Figure 6A).

### Scene Memory Precision

To quantify scene memory precision and the potential influence of route self-similarity, we performed a one-way repeated measures ANOVA, with precision (the MAD of the response error distribution, in meters) for each participant as the dependent variable and route self-similarity (*low*, *int*, and *high*) as the factor. Here, we found a significant effect of route self-similarity on precision, *F*(2, 212) = 28.90, *p* < 0.001, *η*^2^ = 0.214. (Inspection of the QQ plot revealed substantive deviations from normality of the residuals, so we ran a follow-up nonparametric Friedman’s test of differences, which confirmed the effect (*X*^2^ = 111, *p* < 0.001).) A post hoc test for linear trend showed that precision *increased* (lower MAD values) with increasing self-similarity of the route, *F*(1, 212) = 50.40, *p* < 0.001, *η*^2^ = 0.073; OLM(*low*) (*M* = 4.09 m, *SD* = 3.70), STB(*int*) (*M* = 2.40 m, *SD* = 3.18), and JMP(*high*) (*M* = 2.06 m, *SD* = 1.52) (Figure 6B).

### Local Similarity Exploratory Analysis

In this preregistered, exploratory analysis, we sought to characterize the finer-grained relationship between the half-life of each *individual* scene and the precision with which that particular scene was remembered. Would a scene that was relatively similar to its neighbors be remembered with greater, or lesser, precision? To accomplish this, we took a particular scene and calculated its MS-SSIM relative to the other scenes in the route, as a function of distance. (Of course, as in our main analyses, a scene will tend to be quite similar to its neighbors and be less similar as separation increases.) Similarly to our main analysis described above, each of these resulting functions was typically well fit with an exponential decay, from which we could determine that scene’s half-life, yielding a set of 360 half-life values for each route.

We then determined the precision with which each of these scenes was remembered. Since each of the 107 participants only saw 48 scenes from each route (i.e., within the 48-trial block for that route), any particular scene will only have been seen by a subset of participants. We set a minimum that a particular scene had to have been observed by at least 5 participants to be included in this analysis (only 5 of the 1080 (3 * 360) scenes did not meet this threshold, with scenes receiving an average of 12.1 (*SD* = 3.3) observations). We then computed the Kendall correlation between the half-life of each scene within a route and the precision (MAD) with which it was remembered. If scenes with greater local similarity (longer half-lives) are more precisely remembered (lower MAD values), as would be expected based on our main results above, then we should observe a negative correlation. We found weak support for this. The correlation between half-life and MAD was negative and significant for route OLM(*low*): *τ*_*b*_(358) = −0.19, *p* < 0.001, but showed no significant trend for routes STB(*int*): *τ*_*b*_(358) = 0.03, *p* = 0.45 or JMP(*high*): *τ*_*b*_(358) = −0.002, *p* = 0.96.

**Figure 7.**
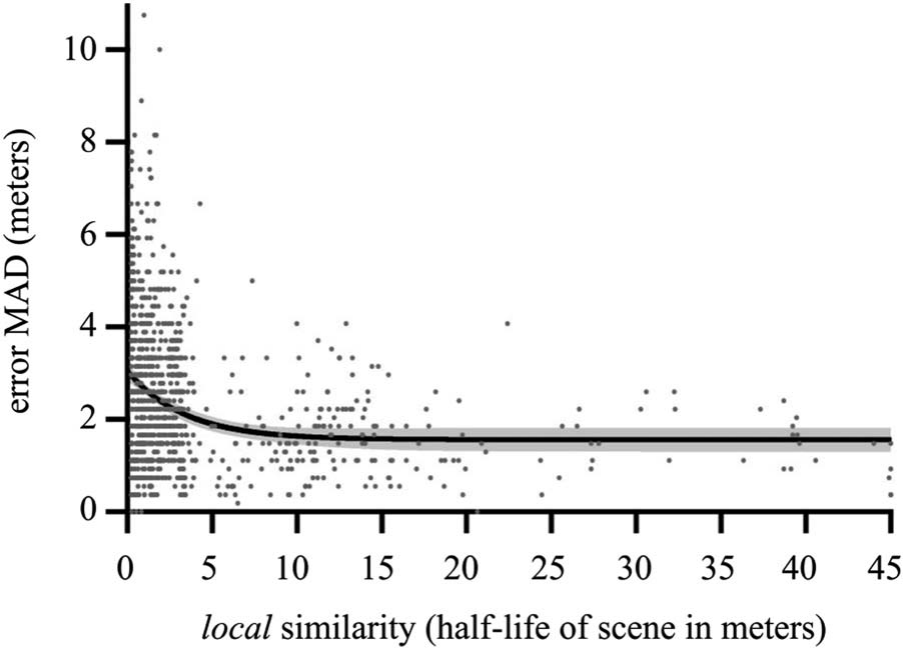
Plot of each scene’s individual half-life (how similar that particular scene was to its neighboring scenes, up to ±45 m) and the precision with which that scene was remembered, shown for all 1080 scenes (360 from each of the three routes). Lower MAD values, as a measure of dispersion, indicate greater precision, and longer half-life indicates greater local similarity. Nonlinear regression (exponential decay) curve is shown with CI band. Consistent with our main results, this exploratory analysis shows that greater local similarity is associated with greater memory precision, here with an asymptotic value of 1.56 m.

Since this potential relationship should hold in general, no matter the particular route, we pooled the three routes to increase power. We found a significant, negative correlation between half-life and MAD computed across all 1080 scenes used in the present study, *τ*_*b*_(1078) = −0.18, *p* < 0.001. To take this exercise a step further we then performed a non-linear regression (exponential decay) on local similarity versus precision. The regression showed that the error MAD drops quickly as a function of half-life, reaching an asymptotic precision of ±1.56 m (Figure 7). (Inspection of the QQ plot revealed substantive deviations from the normality of the residuals, so we performed a robust nonlinear regression (Motulsky & Brown, 2006), which confirmed the results.) While suggestive, we frame this analysis as an exploratory exercise for two reasons: 1) given the inherent variability of natural scenes, *individual* half-life values will be quite volatile (the interquartile ranges of the half-lives for scenes OLM(*low*), STB(*int*), and JMP(*high*) were 0.9 m, 1.65 m, and 10.19 m, respectively) and 2) precision was calculated over a much smaller set of observations (as mentioned above, on average 12.1) than in our main analyses, further increasing variability. That said, consistent with the pattern of results in our main analyses, we found that scenes drawn from *local neighborhoods* with greater self-similarity tend to be remembered with greater precision.

### Gross Mislocalization Analysis

Our main analyses were based on the distribution of errors between the to-be-remembered target scene and the participant’s chosen response. Before those analyses, we removed errors, for each block and participant, that were deemed outliers (> 3 MAD from the median (Leys et al., 2013)). These observations were set aside for this follow-up analysis. We speculated that many of these large errors would be due to gross ‘mislocalizations’, and would be more frequent in routes with greater self-similarity. We found no evidence for this, however. The overall mean rate of mislocalizations was 11.6%, 12.8%, and 13.3% for OLM(*low*), STB(*int*), and JMP(*high*), respectively, and a one-way repeated measures ANOVA with outlier rate as the dependent variable, and with route as the factor was not significant, *F*(2, 212) = 2.87, *p* = 0.06, *η*^2^ = 0.03. We then performed follow-up tests on the accuracy and precision of these large-error responses that paralleled our main analyses (due to (rare) missing values where there were no mislocalizations, we ran mixed effects models instead of one-way repeated measures ANOVA). Here we found the same patterns as in our main results. A mixed effects model with accuracy (median of outliers) and route as the factor showed no effect, *F*(2, 311) = 1.35, *p* = 0.26, *η*^2^ = 0.012, i.e. these responses showed no net forward or backward bias, and were unrelated to route self-similarity: OLM(*low*) (*M* = −3.72 m, *SD* = 29.4), STB(*int*) (*M* = 2.48 m, *SD* = 17.1), JMP(*high*) (*M* = 0.005 m, *SD* = 20.1). Also in line with our main results, a mixed effects model with precision (MAD) of these values as the dependent variable and route and participant as factors showed that precision *was* related to route self-similarity, *F*(2, 205) = 7.98 *p* < .001, *η*^2^ = 0.07, with a follow-up test for linear trend showing that precision increased with increasing route self-similarity, *F*(1, 205) = 15.7, *p* < 0.001, *η*^2^ = 0.07 for each of the three routes: OLM(*low*) (*M* = 19.7 m, *SD* = 12.5), STB(*int*) (*M* = 17.1 m, *SD* = 13.6), and JMP(*high*) (*M* = 13.4, *SD* = 10.4). Since the pattern of these results mirrors those of our main analyses, it is unlikely these large errors stem from lapses, but instead are indeed based, however imprecisely, on scene characteristics.

## DISCUSSION

We sought to quantify the accuracy and precision of memory for natural scenes. Instead of curating a set of images to create a scene space, we took advantage of the ecologically valid, metric space in which scenes occur and are represented in memory: routes. In a delayed estimation, continuous report task, participants were briefly presented with a target scene (drawn from a first-person video of a 90 m outdoor circuit) and then were asked to recall it by moving forward or backward through the (360) scenes that comprised the ‘route loop’. Memory was remarkably accurate, showing no net forward or backward bias. Precision was also very high, with the vast majority of scenes remembered to within a few meters. We found no evidence of boundary extension or contraction. Interestingly too, we found no significant learning effects; this task seems to tap an already well-developed skill.

The present study used isolated images presented on a freestanding display. Future work would benefit from increased visual immersion (e.g., images that occupy a large field of view) presented in an active vision context of navigation. Since the visual system is predisposed to stitch together scenes into larger-scale representations (Hock & Schmelzkopf, 1980) that can then facilitate navigation (Park & Chun, 2009; Robertson et al., 2016); for a review, see Epstein & Baker, 2019), we would expect that such changes would further increase the accuracy and precision of memory for the encountered scenes.

### Aim Small, Miss Small

In a set of pre-registered analyses, we sought to relate the precision of scene memory to the self-similarity of a route (i.e., how rapidly the scenery changes per meter traveled). Contrary to our expectations, we found that memory precision was *higher* for scenes drawn from a more self-similar context. In future work, these results would benefit from further corroboration with a larger set of routes, drawn from different contexts and levels of familiarity (Misra et al., 2018) and that span a larger range of self-similarity values (which, in turn, should be assessed by additional self-similarity metrics beyond MS-SSIM^6^, including those that can account for recognizable landmarks and higher-level scene ‘semantics’). That said, the pattern of results held at both the global level (i.e., overall, scenes from more self-similar routes are remembered with greater precision) and at the local level (i.e., independent of route, a *particular scene* that is more similar to its local neighborhood tends to be remembered with greater precision).

While this result may seem counterintuitive at first, it aligns with models of memory where precision is *regulated* in response to task demands (Orhan et al., 2014; van den Berg et al., 2012). To be clear, it is not that more similar stimuli are easier to distinguish, it’s that the *precision with which a stimulus is remembered* is higher when it is presented in the context of more similar (as opposed to less similar) stimuli. For example, a particular red stimulus will be remembered with greater precision when encoded in the context of other reddish stimuli, as opposed to when it is presented among dissimilar, say, green and yellow, colors (Lin & Luck, 2009; Sanocki & Sulman, 2011). Similarly, experiments on line length and orientation (Sims et al., 2012), and shape (Mate & Baqués, 2009) showed that precision was higher in a condition with lower variance among stimuli. This ‘similarity advantage’ was also found for more complex stimuli. For instance, Jiang et al. (2016) found that memory for faces was better in a condition employing similar faces rather than dissimilar ones. Closest to the present results, in the scene wheel study of Son et al. (2022)—if precision is expressed in terms of the cartesian distance between the target and selected scene within their scene space, i.e., SD of *distance*, instead of SD of degrees of separation along the scene wheel—we can see that when to-be-remembered scenes are drawn from sets with less visual variability, the SD of observers’ errors is lower. In the context of scenes, it seems adaptive to remember a particular scene with greater precision—*aim small, miss small*—when the neighborhood demands it.

### No Boundary Extension or Contraction was Observed

While it was not our focus, the present study provides a thorough test for boundary extension/contraction. 107 participants made judgments about 48 unique scenes drawn from three distinct outdoor environments. In total, 1080 scenes were observed. From trial-to-trial, participants were presented with one of these scenes, then asked to pick it out from among a set of scenes that included versions ‘zoomed’ in or out by very small increments (i.e., those corresponding to forward, or backward, travel at 0.25 m steps). Boundary extension would have been evident as a net ‘backward’ bias in our accuracy measure, while boundary contraction would have been evident as a net ‘forward’ bias in our accuracy measure. As reported above, we found very high accuracy, i.e. no net bias. This result adds to the increasing skepticism about the ubiquity of boundary extension, consistent with recent explorations of the effect which found evidence for both extension *and* contraction, with the effect idiosyncratically related to the nature of the images in question (Bainbridge & Baker, 2020; Lin et al., 2022).

### Ecological Validity

A quantitative characterization of scene memory requires parameterization of stimuli, but there is an indefinitely large number of potential dimensions along which to manipulate scene properties. However, not all scene variations are equally likely or behaviorally relevant (Felsen & Dan, 2005; Hayhoe & Rothkopf, 2011). Within the unconstrained ‘scene space’, there are ecologically valid (Brunswik, 1955) lower-dimensional manifolds defined by the visual consequences of natural events: weather conditions, time of day, seasonal variations, growth and decay, or here, a literal path connecting one place to another. Of course, there are many visual consequences to travel along a route, including geometric changes based on visual optics, but also the presence and position of objects and landmarks that interact with expectations about scene gist (Smith & Loschky, 2019) and content (Võ, 2021). Future work can tease them apart, but the point of the present study was to explore a framework that *links* these various factors.

## CONCLUSION

Is scene memory good or bad? This is a question that cannot be answered without context. Memory for natural scenes, in the ecologically valid context of a route, is remarkably good: participants can remember a scene to within a few meters of its actual location, more than adequate for a walk in the park.

## ACKNOWLEDGMENTS

We thank Gaeun Son for generously sharing JavaScript code and advice for stimulus presentation. We would also like to thank Prof. Zsuzsa Kaldy for critical suggestions and feedback.

## AUTHOR CONTRIBUTIONS

L. W.: Data curation, Formal analysis, Investigation, Project administration, Software, Validation, Visualization, Writing – original draft. Y. L.: Data curation, Software, Writing – review & editing. E. B.: Conceptualization, Data curation, Formal analysis, Investigation, Methodology, Project administration, Resources, Supervision, Validation, Visualization, Writing – original draft, Writing – review & editing.

## OPEN SCIENCE PRACTICES

This study was preregistered. Preregistration, data, and materials are shared on OSF (Blaser & Westebbe, 2022): osf.io/a7mkt.

## Notes

^1^ Indeed, competitive memorizers exploit this intrinsic memory for routes with the *Method of Loci* (aka Memory Palace or Journey Method) to encode and recall large sets of, for instance, numbers, playing cards, or words (Roediger, 1980) by mentally placing to-be-remembered information in specific scenes along a known route: e.g., Yanjaa Wintersoul’s 2018 World Record of memorizing 145 random words in 5 minutes. Importantly, this is a method that only requires a brief period of instruction (Bass & Oswald, 2014) and utilizes pre-existing hippocampal networks for spatial memory (Maguire et al., 2003).^2^ Of course, navigation depends upon - and is influenced by - much more than just scene memory, including knowledge of heading, required turns, and terrain, along with (often systematically biased) estimates of distance and time to the goal location (Brunec et al., 2017). For *visual* navigation, better scene memory should facilitate navigation and we would further predict, based on our findings here (see Results), that aspects of navigation (memory for a particular turn, say) may be regulated according to task demands and thereby improved within environments that are visually more self-similar.^3^ Including Canada (1), Chile (1), Czech Republic (1), France (2), Germany (2), Greece (6), Hungary (5), Ireland (2), Italy (9), Mexico (17), Poland (22), Portugal (15), South Africa (6), Spain (8), UK (10).^4^ By convention, all MAD values reported in this manuscript were multiplied by a scaling factor of 1.4826 to render them consistent estimators of standard deviation (Leys et al., 2013).^5^ To corroborate this ranking we performed three follow-up tests, using variations on our preregistered approach. First, to help ensure the ranking was not driven by the metric of self-similarity itself, and for consistency with previous work (Son et al., 2022), we determined self-similarity based on simple pixel-wise image correlation. The resulting rank ordering was the same as determined by MS-SSIM. Then, to help ensure the ranking was not driven by the choice of basing it on half-life, we instead simply computed the *overall average* self-similarity for a route (i.e., the overall mean of all pairwise similarity values, as seen in the heatmaps in Figure 4). This also gave the same ranking of the routes. Finally, we returned to our main analyses but applied them to *alternate takes* of each route (i.e. videos independently collected along each of the same routes). The ranking of these alternate takes matched that of the main takes. Please see Supplemental Tables 1 and 2 on OSF (Blaser & Westebbe, 2022).^6^ Indeed, any conclusions about the relationship between self-similarity and scene memory can only be as strong as our trust in the self-similarity measure itself (Venkataramanan et al., 2021). While an in-depth comparison is beyond the scope of this study, we have added information in Supplemental Tables 1 and 2 on OSF (Blaser & Westebbe, 2022) to give insight into which image aspects drove the self-similarity rankings.

## Supplementary Material


